# Identification, Selection, and Enrichment of Cardiomyocyte Precursors

**DOI:** 10.1155/2013/390789

**Published:** 2013-06-18

**Authors:** Bianca Ferrarini Zanetti, Walter José Gomes, Sang Won Han

**Affiliations:** ^1^Department of Biophysics, Gene Therapy Investigation Center, Universidade Federal de São Paulo, Rua Mirassol 207, 04044-010 São Paulo, SP, Brazil; ^2^Department of Surgery, Universidade Federal de São Paulo, São Paulo, SP, Brazil

## Abstract

The large-scale production of cardiomyocytes is a key step in the development of cell therapy and tissue engineering to treat cardiovascular diseases, particularly those caused by ischemia. The main objective of this study was to establish a procedure for the efficient production of cardiomyocytes by reprogramming mesenchymal stem cells from adipose tissue. First, lentiviral vectors expressing neoR and GFP under the control of promoters expressed specifically during cardiomyogenesis were constructed to monitor cell reprogramming into precardiomyocytes and to select cells for amplification and characterization. Cellular reprogramming was performed using 5′-azacytidine followed by electroporation with plasmid pOKS2a, which expressed Oct4, Sox2, and Klf4. Under these conditions, GFP expression began only after transfection with pOKS2a, and less than 0.015% of cells were GFP^+^. These GFP^+^ cells were selected for G418 resistance to find molecular markers of cardiomyocytes by RT-PCR and immunocytochemistry. Both genetic and protein markers of cardiomyocytes were present in the selected cells, with some variations among them. Cell doubling time did not change after selection. Together, these results indicate that enrichment with vectors expressing GFP and neoR under cardiomyocyte-specific promoters can produce large numbers of cardiomyocyte precursors (CMPs), which can then be differentiated terminally for cell therapy and tissue engineering.

## 1. Introduction

Cardiovascular diseases are the number one cause of death worldwide. In 2008, approximately 17.3 million people died from cardiovascular diseases, representing 30% of global deaths [1]. Cardiac muscle tissue lost by ischemia is replaced by scar tissue, which overloads cardiovascular activity and can lead to heart failure in the long term [2]. The development of new drugs, equipment, and surgical techniques has ameliorated these cardiac symptoms, but the risk of death associated with heart failure is still high, and long-term treatments cause high economic impact [3]. 

One way to circumvent this pathological progression is to replace the damaged tissue with functional tissue. This task can be performed by transplanting *in loco* cardiomyocytes or cardiomyocyte stem cells or precursor cells. If the injured area is too large, then these cells can be placed in a scaffold and transplanted into the heart after removal of fibrous tissue [3, 4]. However, to perform these processes, a large amount of the patient's cardiomyocytes is necessary, which is one of the main challenges today. In theory, these cells can be obtained from adult stem cells residing in the heart, but there are technical difficulties in obtaining them in sufficient amounts. For example, cardiac biopsy from a sick heart is a highly risky procedure [5]. 

On the other hand, embryonic stem cells (ESCs) seem to be a good source of functional cardiomyocytes [6]; however, several biological issues related to teratoma formation, immunological rejection, and chromosomal instability, as well as the ethical issue of the source of ESCs, are highly concerning [7]. Induced pluripotent stem cells [8] can overcome the ethical issue, but the concerns related to the ESC properties remain.

Mesenchymal stem cells (MSCs) have emerged as an alternative source of cardiomyocytes and other cell types because these cells can be obtained in large amounts from adult fat tissue and bone marrow [9, 10]. However, several methods of cell differentiation have led to a small percentage of cardiomyocyte-like cells from MSCs. It is important to note that, in these papers, the authors characterized the differentiated cardiomyocytes with a small number of cardiomyocyte markers by immunocytochemistry and/or RT-PCR, which cannot represent true cardiomyocytes [11–15]. Additional analyses, such as electrophysiology, will be necessary to validate these cells. 

There are at least three critical issues to be considered in order to produce cardiomyocytes on a large scale using stem cells: the efficiency of differentiation in cardiomyocytes is relatively low [16], these differentiated cells have variable phenotypes [12, 14], and the expansion of cardiomyocytes is difficult because mature cardiomyocytes are senescent [17]. These issues can be overcome if the MSCs that are destined to differentiate into cardiomyocytes can be identified and selected before differentiating terminally into cardiomyocytes, as these selected precardiomyocytes, or cardiomyocyte precursors (CMPs), should be more homogeneous than terminally differentiated cardiomyocytes and should possess the capacity to divide. 

In this study, we describe a method of enrichment of CMPs from MSCs to facilitate cell multiplication and differentiation into cardiomyocytes later. This approach is based on the use of vectors with cardiomyocyte-specific gene promoters, such as *ATP2A2*, *GJA1*, and *NPPA*, to direct the expression of neoR and GFP in MSC cultures undergoing differentiation into cardiomyocytes. 

The *ATP2A2 *gene encodes for SERCA2a protein (NG_007097.2), a sarcoplasmic ATPase involved in the regulation of contraction and relaxation cycle. This gene is activated at the heart chambers during heart development and is expressed over adult life [18].

The *GJA1* gene encodes for connexin 43 (NG_008308.1), a gap junction protein that is necessary for electrical coupling of cardiomyocytes. The *GJA1* expression starts early in the embryonic development and is present in adult organs [19].

The *NPPA* gene encodes for natriuretic peptide A (NG_012926.1), a protein implicated in the control of extracellular fluid volume and electrolyte homeostasis. Its expression starts at linear heart tube formation, thus before GJA1 expression but earlier than *ATP2A2* expression. The expression of *NPPA* is related to the maturation of cardiomyocytes and its expression in adult phase occurs only at cardiac atria [20].

The use of different promoters allows for the selection of cells at different stages of cardiomyocyte differentiation, which can then be compared with respect to their efficiency of proliferation, ability to differentiate into cardiomyocytes, and cell quality.

## 2. Materials and Methods 

### 2.1. Construction of Lentivectors with Cardiomyocyte-Specific Promoters

The neoR cDNA was excised from the pDsRed-Monomer-C1 vector (Clontech, Mountain View, CA, USA) with *Avr*II (all restriction enzymes were purchased from NEB, Ipswich, MA, USA, and Fermentas, Hanover, MD, USA), treated with Klenow polymerase (NEB), and inserted into the *Sma*I site of pIRES2-EGFP (Clontech). This final vector was named pNeoR-IRES2-EGFP. 

The *ATP2A2* promoter (585 bp) was obtained by the digestion of the vector pGL2SER-263 ([21]; kindly provided by Dr. Kenneth R. Boheler, NIH, USA) with the restriction enzymes *Kpn*I and *Xba*I. The fragment *ATP2A2* was treated with Klenow and inserted at the *Sma*I  site of the pBSK plasmid (Stratagene, La Jolla, CA, USA). pBSK-ATP2A2 and pNeoR-IRES2-EGFP were digested with* Eco*RI  and  *Bam*HI and ligated. The product of the last reaction was digested with *Nhe*I and *Bsr*GI to obtain the ATP2A2-neoR-IRES2-EGFP cassette; this cassette was then inserted into the lentiviral vector pLL3.7 [22], which was previously digested with *Xba*I and *Bsr*GI. The final vector was named pLL-ANG.

The *GJA1* (503 bp) and *NPPA* promoters (577 bp) were amplified by PCR from human genomic DNA from HEK293T cells using the following primers (Integrated DNA Technologies, Coralville, IA, USA): GJA1p_Forward 5′-TTGAATTCTGGTTATATGCTTCCCCACC, GJA1p_Reverse 5′-AAGTCGACAAGTGATTGAACTCCTTGGAGG, NPPAp_Forward 5′-TTGAATTCATGAGGCAGGTGTGAGGC, and NPPAp_Reverse 5′-TTGTCGACCCACTGCTTGCTGCTCTG. These primers were designed based on data from the literature [23–26], and *Eco*RI and *Sal*I sites were included in the forward and reverse primers, respectively. The amplification conditions were 95°C for 3 min followed by 35 cycles of 95°C for 30 s, 52°C (*GJA1*) or 55°C (*NPPA*) for 60 s, and 72°C for 60 s, and finally 72°C for 7 min (Mastercycler Gradient 5341, Eppendorf, Hamburg, Germany). The DNA fragments containing the promoter sequences were digested with *Eco*RI and *Sal*I and inserted into pNeoR-IRES2-EGFP, which was previously digested with the same enzymes. The GJA1-neoR-IRES2-EGFP and NPPA-neoR-IRES2-EGFP cassettes were excised with *Xho*I and *Bsr*GI enzymes and inserted into the lentiviral vector pLL3.7 [22], which was previously digested with the same enzymes. The final vectors were named pLL-GNG and pLL-NNG, which contained the *GJA1* and *NPPA* promoters, respectively.

A control vector containing the CMV promoter was also constructed by the ligation of pNeoR-IRES2-EGFP and pLL3.7 vectors digested with *Nhe*I and *Bsr*GI. This vector was named pLL-CNG. All final vectors were analyzed by DNA sequencing.

### 2.2. Cell Culture

The cell lineages HEK293T (human embryonic kidney cell line) [27], NIH3T3 (murine fibroblast cell line) [28], and H9C2 (rat cardiomyoblast cell line) [29] were maintained in Dulbecco's Modified Eagle Medium (DMEM, GIBCO, Auckland, New Zealand) supplemented with 10% fetal bovine serum (FBS, GIBCO), 0.2 mM glutamine (Glutamax-1, GIBCO), 10 U/mL penicillin, and 10 *μ*g/mL streptomycin (GIBCO). The human endothelial cell line ECV304 [30] was maintained in DMEM/F12 medium (Cultilab, Campinas, SP, Brazil) supplemented with 10% FBS, 0.2 mM glutamine (Glutamax-1, GIBCO), 10 U/mL penicillin, and 10 *μ*g/mL streptomycin (GIBCO).

Mesenchymal stem cells from adipose tissue (ADSCs) were isolated from human lipoaspirate samples according to Zuk et al. [31]. Briefly, these cells were washed extensively with PBS (137 mM NaCl, 2.7 mM KCl, 10 mM Na_2_HPO_4_, and 2 mM KH_2_PO_4_; pH 7.4) and incubated with 1 mg/mL collagenase type I at 37°C for 10 minutes. Collagenase was neutralized by addition of Minimum Essential Medium (*α*-MEM, GIBCO) supplemented with 10% FBS, 0.2 mM glutamine, 10 U penicillin, and 10 *μ*g streptomycin. The cells were centrifuged at 800 ×g for 10 minutes. The precipitated cells were suspended at a concentration of 2 × 10^6^ cells/mL in *α*-MEM supplemented with 10% FBS, 0.2 mM glutamine, 10 U/mL penicillin, and 10 *μ*g/mL streptomycin. The cells were maintained in this medium, which was replaced with fresh medium every 2 to 3 days. 

### 2.3. Viral Production and Titration

Viral vectors were produced following a method described by Barde et al. [32]. Briefly, 2 × 10^6^ HEK293T cells were plated on each 10 cm^2^ plate containing DMEM with 10% FBS, 0.2 mM glutamine, 10 U penicillin, and 10 *μ*g streptomycin. Four 10 cm^2^ plates were used to produce each batch of viral vector. One day after plating, the medium was replaced with fresh medium, and 20 *μ*g of transfer vector, 5 *μ*g pRSV-REV, 10 *μ*g pMDLg/pRRE, and 6 *μ*g pCi-VSVG were used to transfect each plate of HEK293T cells by the calcium phosphate coprecipitation method. The culture supernatant was collected after 24 and 48 hours, filtered using a 0.45 *μ*m syringe (Millex HV 0.45 *μ*m, Millipore, Massachusetts, USA), and concentrated by centrifugation at 14,000 rpm at 4°C for 2 hours in a sucrose gradient (Sorvall RC 5C Plus, SA600 rotor, Thermo Scientific, North Carolina, USA). The concentrated materials were stored at −80°C until use.

pLL-CNG virus was titrated based on the method described by Barde et al. [32]. In a 24-well plate, 2 × 10^4^ HEK293T cells were plated in DMEM with 10% FBS, 0.2 mM glutamine, 10 U penicillin, and 10 *μ*g streptomycin. After 24 hours, various concentrations of virus were added to each well in the presence of 8 *μ*g/mL Polybrene (Sigma-Aldrich, Milwaukee, USA). After 48 hours, the cells were analyzed by fluorescence microscopy (IX70 inverted microscope, Olympus, Tokyo, Japan). This experiment was repeated at least three times, and the titer was calculated by the mean of the titers plotted in the linear phase of the graph according to the following equation:

(1) Titer (TU/mL) =%GFP positive cells×total number of cellsvirus volume (mL),

TU: transduction units.

### 2.4. Validation of Cardiomyocyte-Specific Promoters

**Figure 1:**
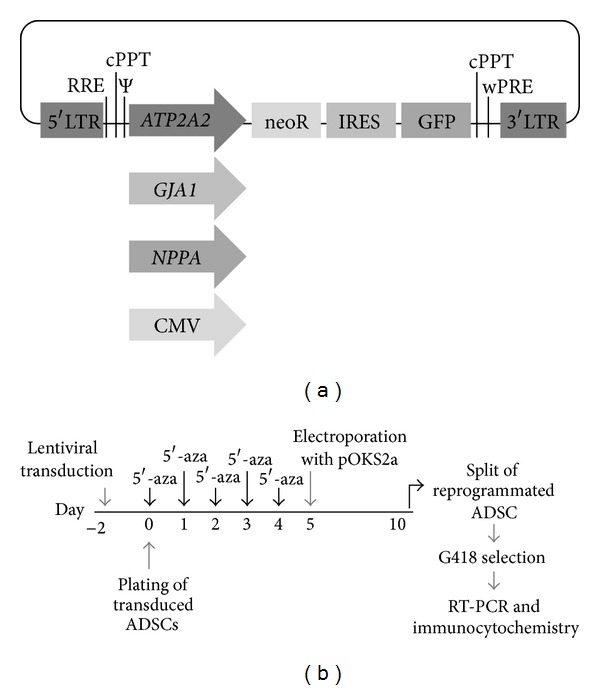
Lentiviral vectors and timeline of ADSC reprogramming. (a) Lentiviral vectors containing different cardiomyocyte-specific promoters. LTR: long terminal repeat; RRE: Rev-responsive element; Ψ: packaging signal; *ATP2A2/GJA1/NPPA/CMV*: promoters; neoR: G418 resistance gene; IRES: internal ribosome entry site; GFP: green fluorescent protein; cPPT: central polypurine tract; WPRE: Woodchuck hepatitis virus posttranscription regulatory element. (b) Timeline of the ADSC reprogramming to the formation of CMPs. Lentiviral transduction was performed with pLL-CNG, pLL-ANG, pLL-GNG, and pLL-NNG vectors. 5′-aza: 5′-azacytidine.

To validate the cardiomyocyte specificity of the *ATP2A2*, *GJA1*, and *NPPA* promoters, H9C2, HEK293T, NIH3T3, and ADSC cells were transduced with the lentivectors constructed in Section 2.1 and shown in Figure 1. For the transduction of ADSC and H9C2 cells, 4 × 10^4^ cells were plated per well in a 6-well plate; for HEK293T and NIH3T3 cells, 2 × 10^4^ cells were plated per well in a 24-well plate. The next day, the medium was replaced with fresh medium containing 8 *μ*g/mL Polybrene, and 10 *μ*L of viral solution was added. 

The expression of GFP was monitored for 9 days using an IX70 inverted fluorescence microscope (Olympus). The transduced cells were selected by adding 1 mg/mL G418 (Invitrogen, Carlsbad, CA, USA) to the medium, which was replaced with fresh medium containing 1 mg/mL G418 every 2 to 3 days for 10 days. The selected cells were plated on coverslips placed in 24-well plates at 1 × 10^3^ cells per well and cultured using the same cell culture conditions described previously. After 24 hours, the medium was siphoned off carefully, the cells were fixed with 4% paraformaldehyde, and epifluorescence images were acquired for analysis (BX51 fluorescent microscope, Olympus).

### 2.5. Reprogramming, Selection, and Characterization of ADSCs

The ADSCs were plated in 6-well plates at a concentration of 5 × 10^4^ cells per well. The next day, the cells were transduced with 20 *μ*L of virus in the presence of 8 *μ*g/mL of Polybrene. After 48 hours, the cells were transferred into T75 flasks at a density of 2 × 10^5^ cells per flask, and 5′-azacytidine (Sigma-Aldrich) was added at a final concentration of 10 *μ*M for 96 hours with daily medium changes. The reprogramming was immediately followed by electroporation (BTX, Electro Square Porator ECM830, Holliston, USA) with the pOKS2a plasmid [33]. The 5′-azacytidine-treated cells were detached and suspended at a concentration of 5 × 10^5^ cells in 50 *μ*L Spinner's modification of Eagle's minimum essential medium (SMEM, Life Technologies, Paisley, UK) with 8 *μ*g of pOKS2a. The electroporation machine was programmed to 12 pulses of 150 V and 150 *μ*s duration.

After 5 days, the cells were split 1 : 3, and 1 mg/mL G418 was added to the medium for selection for 10 days; the medium was replaced with fresh medium every 2 to 3 days. 

For RT-PCR analysis, total RNA was extracted from these cells using the RNeasy Mini kit (Qiagen, Hilden, Germany), and cDNA was synthesized using the High Capacity cDNA Reverse Transcription Kit (Applied Biosystems, California, USA) following instructions provided by the manufacturers. The following primers were used for RT-PCR: *MYH7*, which codes for βMHC (GGCAAGACAGTGACCGTGAAG and CGTAGCGATCCTTGAGGTTGTA); *GJA1*, which codes for connexin 43 (AGGCGTGAGGAAAGTACCAA and ACACCTTCCCTCCAGCAGTT); *ATP2A2*, which codes for SERCA2a (GGTGCTGAAAATCTCCTTGC and ATCAGTCATGCACAGGGTTG); *GATA4*, which codes for the protein of the same name (GAGTAAACAAGAGCCTAGAGCCC and AGAAAACGACGGCAACAACG); *MEF2C*, which codes for the protein of the same name (GAACAATCCCGGTGTGTCAGGA and CACCCAGTGGCAGCCTTTTACA); and *GAPDH*, which codes for glyceraldehyde 3-phosphate dehydrogenase (ACCACAGTCCATGCCATCAC and TCCACCACCCTGTTGCTGTA) (Bioneer, Alameda, USA). The amplification conditions were 95°C for 5 min followed by 35 cycles of 95°C for 45 s, 45 s for extension [(*ATP2A2*)/51°C; (*GJA1*)/54°C; (*GATA4*)/55°C; (*MYH7*, *GAPDH*); 58.2°C (Mef2c)] and 72°C for 45 s, and finally 72°C for 7 min (Mastercycler Gradient 5341, Eppendorf). 

The RNA extracted from a human heart biopsy was used as a positive control. The biopsy was acquired with the consent of a patient undergoing cardiac surgery after approval of the protocol by the ethics committee of UNIFESP (CEP-UNIFESP 03/2810). RNA from ECV cell line was used as a negative control. Total RNA was extracted using TRIzol (Invitrogen) following instructions provided by the manufacturer. cDNA was synthesized using the High Capacity cDNA Reverse Transcription Kit (Invitrogen).

In addition to RT-PCR, these cells were characterized by immunocytochemistry using anti-desmin, anticardiac troponin T, antismooth muscle actin, and anti-von Willebrand factor antibodies (Millipore MAB1693, Millipore MAB3430, Dako M0851, and Dako A0082, resp.). The selected cells were plated on coverslips placed in 24-well plates at 1 × 10^3^ cells per well. After 48 hours, the medium was siphoned off carefully, and the cells were fixed in cold acetone for 10 minutes at −20°C, washed with PBS, and blocked with 50 mM NH_4_Cl, pH 8.0 solution for 15 minutes followed by washing and blocking with PBS-10% BSA for 30 minutes. The cells were washed again and incubated overnight at 4°C with the primary antibodies diluted 1 : 100 in PBS-1% BSA containing 0.01% Triton X-100, 0.01% Tween 20, and 0.01% gelatin. The next day, the samples were washed and incubated with biotin-labeled polyclonal rabbit anti-mouse IgG secondary antibody (Dako, Glostrup, Denmark) diluted 1 : 100 in PBS for 1 hour at room temperature in the dark. After washing with PBS, the samples were incubated with Streptavidin Alexa Fluor 594 Conjugate (Life Technologies, Grand Island, NY, USA) diluted 1 : 500 in PBS for 1 hour at room temperature under light protection. The samples were washed and incubated with DAPI (Life Technologies, Eugene, OR, USA) diluted 1 : 1000 for 15 minutes at room temperature in the dark. The coverslips were mounted with Fluoromount (Sigma-Aldrich, Milwaukee, WI, USA) and analyzed using a BX51 fluorescent microscope.

## 3. Results and Discussion

 To identify and select CMPs to produce cardiomyocytes in large numbers at a later stage, lentiviral vectors were constructed containing an expression cassette with the GFP and neoR genes under the control of cardiomyocyte-specific promoters *ATP2A2*, *GJA1*, and *NPPA* (Figure 1). Because the GFP and neoR genes were under the control of the same cardiomyocyte-specific promoters, the green fluorescence emitted by the transfected cells should indicate that these cells were undergoing reprogramming into cardiomyocytes and that they could be selected for G418 resistance for further characterization, expansion, and terminal differentiation. 

In this study, a vector with the CMV promoter, which is a strong and constitutively active promoter [34], was constructed using the same backbone as the previously vectors as a control. The viral titer of this vector, named pLL-CNG, using HEK293T cells was 2.3 × 10^8^ TU/mL. HEK293T cells, which are easy to culture and expand, have efficient machinery for protein synthesis, which allows for high viral and GFP production [35]. For this reason, this cell line has been used as a standard cell line for lentiviral production and viral titer determination [36].

To determine the viral titers of the vectors carrying cardiomyocyte-specific promoters, we used the murine cardiomyoblast cell line H9C2 because no GFP fluorescence was seen using HEK293T cells, which is an important proof of the specificity of these promoters. Viral titers were 7.9 × 10^5^ TU/mL, 2.6 × 10^5^ TU/mL, 7 × 10^5^ TU/mL, and 1.5 × 10^5^ TU/mL for pLL-CNG, pLL-ANG, pLL-GNG, and pLL-NNG, respectively. The viral titers of pLL-CNG in H9C2 were three orders of magnitude lower than in HEK293T cells. This result allows us to infer that viral vectors containing cardiomyocyte-specific promoters should provide titers that are three orders of magnitude higher in HEK293T cells than the other cell types, and this magnitude of titer is typical in this cell line [37]. 

pLL-derived vectors are pseudotyped with VSV-G, which recognizes cell membrane phospholipids for self-internalization [38] and allows for the transduction of a large number of cell types efficiently. However, the vector titer also depends on the promoter activity. The CMV promoter is a well-known, constitutively active promoter, but its activity varies among different cell types [37]. The viral titers of the aftermentioned vectors were consistently the same order of magnitude in our preparations (not shown), which demonstrates the reproducibility of vector production.

**Figure 2:**
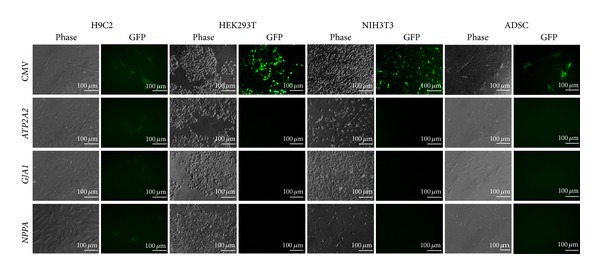
GFP gene expression by different cell types transduced with lentivectors containing cardiomyocyte-specific promoters. A lentivector containing CMV promoter was used as a positive control. Cell images were acquired 48 hours after vector transduction.

 To validate the cardiomyocyte specificity of the constructed vectors, these vectors were tested in cells from different tissues of origin: NIH3T3 from fibroblasts, HEK293T from kidney, ADSCs from fat, and H9C2 from heart. As discussed previously, HEK293T cells transduced with pLL-CNG had many GFP-positive cells with strong fluorescence intensity (Figure 2). The GFP-positive cells were also present in H9C2, ADSC, and NIH3T3 cells transduced with pLL-CNG, but the number of cells and the fluorescence intensity were much lower than in HEK293T cells. On the other hand, lentivectors constructed with cardiomyocyte-specific promoters functioned only in H9C2 cells, and no GFP-positive cells were found in HEK293T, NIH3T3, or ADSC cells (Figure 2). The number of GFP-positive cells and the fluorescence intensity were similar to the values observed in cells transduced with pLL-CNG. Therefore, these vectors were highly specific to cardiomyocytes. 

**Figure 3:**
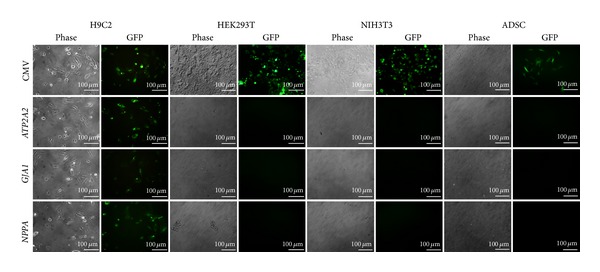
GFP gene expression by different cell types transduced with lentivectors containing cardiomyocyte-specific promoters and selected with G418. A lentivector containing CMV promoter was used as a positive control. Cell images were acquired 10 days after G418 selection.

Another goal of this work was to demonstrate that these GFP-positive cells could be selected for G418 antibiotic resistance for large-scale amplification at a later stage. The H9C2 cells transduced with cardiomyocyte-specific vectors were resistant to G418, and most of the selected cells, if not all, were GFP positive (Figure 3). In contrast, the non-heart-derived NIH3T3, HEK293T, and ADSC cells transduced with cardiomyocyte-specific promoters did not survive in the presence of G418. All cell types transduced with pLL-CNG were resistant to G418 and positive for GFP. These selected cells did not exhibit prolonged doubling times even after a few months of observation (not shown). These results demonstrate the specificity, selectivity, and expandability of the G418-resistant cells.

Once the correct functioning of the cardiomyocyte-specific vectors was verified, these vectors were tested in ADSCs, which were conditioned to differentiate into cardiomyocytes. A small population of ADSCs can differentiate into cardiomyocytes when incubated with 5′-azacytidine [11, 12, 14, 15]. To enforce this differentiation, we transfected these cells with Yamanaka factors [8] soon after incubation with 5′-azacytidine (see Section 2). Approximately 0.007%, 0.009%, and 0.004% of ADSCs were GFP positive after transduction with pLL-ANG, pLL-GNG, and pLL-NNG, respectively. 

Note that the percentages of GFP-positive cells that were undergoing reprogramming to form cardiomyocytes were very small. These numbers are much smaller than those seen in the literature [11, 12, 14, 15]. None of the previous papers used cardiomyocyte-specific promoters with a reporter gene to monitor the reprogramming process, and most of them evaluated some cardiomyocyte markers by immunocytochemistry to validate the positivity of cardiomyocytes. As the methods used to identify cardiomyocytes are different among the different papers, a quantitative comparison is difficult; however, the promoters used here are activated specifically during different stages of cardiomyogenesis, so it is likely that what we saw by GFP fluorescence were cardiomyocyte precursor cells at different stages of differentiation, and this fact can explain such low numbers of GFP-positive cells.

**Figure 4:**
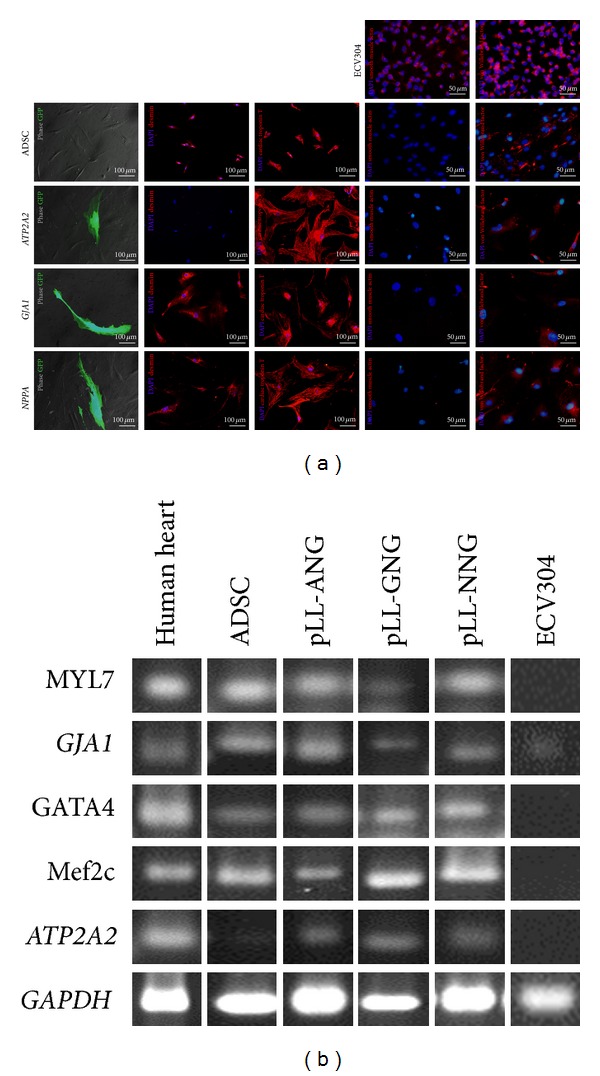
Characterization of CMPs by immunocytochemistry and RT-PCR. (a) ADSCs transduced with lentivectors before G418 selection (first column). These cells were selected with G418, expanded, and stained with anti-desmin (second column), anticardiac troponin T (third column), antismooth muscle actin (fourth column), and anti-von Willebrand factor (fifth column) antibodies. The nontransduced ADSCs were used as control (first line) and ECV304 cells were used as positive control for smooth muscle actin and von Willebrand factor staining. (b) RT-PCR of the selected cells for the cardiomyocyte-specific genes. The *GAPDH* gene was used as control. The RNAs extracted from a human heart and ECV were used as positive and negative controls, respectively.

Finally, these GFP-positive ADSCs were selected for G418 resistance for expansion and further characterization. The selected ADSCs were amplified in culture for months without variability in the doubling time, which indicates that these cells are not completely differentiated into cardiomyocytes. However, the morphology changed over time from fibroblast-like cells to flattened, cubic cells. These reprogrammed and selected ADSCs were positive for troponin T (Figure 4(a)), had flat and cuboid shapes, and contained intracellular fibers, all characteristics of cardiomyocytes [39]. However, the desmin staining pattern, as assessed by immunocytochemistry, was notably different among the reprogrammed cells (Figure 4(a)); specifically, the ADSCs modified with pLL-GNG and pLL-NNG were positive for desmin, but the pLL-ANG-modified cells were not. Unlike the labeling with troponin T, the labeling with desmin showed a homogeneous pattern across the whole cell area, without evidence of fibers. None of the selected cells were positive for smooth muscle actin, a typical marker for smooth cells, but they were positive for von Willebrand factor, a typical marker for endothelial cells, including the nondifferentiated ADSC. The expression of von Willebrand factor in ADSC has not been reported yet, but in hematopoietic mesenchymal stem cells its expression was detected by RT-PCR [40]. Further characterization of these cells by RT-PCR showed positivity for five genetic markers of cardiomyocytes (Figure 4(b)). These data indicate that the reprogrammed and selected ADSCs had cardiomyocyte characteristics, albeit with significant differences among them because these cells are still undergoing the reprogramming process. 

The RT-PCR results showed that the ADSCs were also positive for most of the cardiomyocyte markers, except for *ATP2A2* (Figure 4(b)). ADSCs and cardiomyocytes share several similarities [12–14, 41–43], suggesting that ADSCs really do have the potential to differentiate into cardiomyocytes. This fact implies that the number of cardiomyocytes found after MSC differentiation in other studies might have been overstated because some of the cardiomyocyte markers detected might have been from the source and not the differentiated cells. Consequently, the small numbers of GFP-positive cells found in our study might be closer to the real number of cardiomyocytes in these cultures than the numbers found by others. 

In conclusion, we demonstrated that lentivectors expressing GFP and neoR genes under the control of cardiomyocyte-specific promoters can identify MSCs undergoing reprogramming into cardiomyocytes, and the CMPs can be selected for G418 resistance for expansion and terminal differentiation in cardiac cell-based therapies.
